# Reversible Dilated Cardiomyopathy Associated With Herpes Simplex Virus Infection: A Case Report

**DOI:** 10.7759/cureus.76174

**Published:** 2024-12-22

**Authors:** Tânia F Mendes, Bárbara F Silva, Nuno A Sousa

**Affiliations:** 1 Internal Medicine, Hospital Vila Franca de Xira, Vila Franca de Xira, PRT

**Keywords:** acute heart failure, antiviral therapy, dilated cardiomyopathy, herpes simplex virus, viral myocarditis

## Abstract

Dilated cardiomyopathy (DCM) is a serious condition often leading to acute heart failure (HF), with diverse etiologies including viral myocarditis. This report details a case of reversible DCM in a 34-year-old male who presented with symptoms of acute HF. Diagnostic workup revealed biventricular dilation with severe systolic dysfunction and serology confirming herpes simplex virus infection. The patient was treated with standard HF therapy and colchicine, leading to significant clinical improvement. Despite the positive viral serology, antiviral therapy was not initiated due to the patient’s marked recovery, underscoring the controversial role of antivirals in viral myocarditis-associated DCM. At a one-year follow-up, the patient demonstrated substantial recovery of cardiac function. This case highlights the potential for favorable outcomes with comprehensive management, even in the absence of antiviral therapy, and emphasizes the need for individualized treatment strategies in DCM related to viral myocarditis.

## Introduction

Dilated cardiomyopathy (DCM) is a condition characterized by dilation and impaired contraction of the left or both ventricles, leading to systolic dysfunction and acute heart failure (HF). It is a major cause of HF and is associated with significant morbidity and mortality [1]. The etiology of DCM is diverse, encompassing genetic, toxic, metabolic, and infectious causes. Among these, viral myocarditis is a recognized precursor to DCM, particularly when the viral infection leads to persistent myocardial inflammation and subsequent myocardial remodeling [2,3].

Viral myocarditis, often caused by enteroviruses (e.g., Coxsackievirus B), adenoviruses, and herpesviruses, can trigger an inflammatory response in the myocardium. This response can result in direct myocardial injury, autoimmune reactions, and, eventually, the development of DCM if the inflammation persists or if there is significant myocardial damage. The progression from acute viral myocarditis to DCM is complex, involving both direct viral damage to cardiac myocytes and the host’s immune response, which can lead to chronic inflammation and fibrosis [4,5]. Documented arrhythmias have been reported more commonly with human immunodeficiency virus (HIV) myocarditis than other more common infections such as adenovirus, parvovirus B19, human herpes virus 6, and enterovirus [6].

The treatment of DCM, particularly when associated with viral myocarditis, is complex and multifaceted. Current guidelines advocate for standard HF management, which includes the use of angiotensin-converting enzyme inhibitors (ACEi), sodium-glucose cotransporter-2 (SGLT2) inhibitors, beta-blockers, diuretics, and, in select cases, aldosterone antagonists [2,6,7].

There is substantial evidence supporting the efficacy of beta-blockers in non-ischemic dilated cardiomyopathy (NIDCM), with carvedilol emerging as a particularly effective option. In contrast, the role of ACEi in NIDCM is less clearly defined, highlighting the need for further studies specifically targeting this condition to optimize treatment strategies for this unique condition [8].

In instances where a viral etiology is suspected or confirmed, antiviral treatment may be warranted, particularly in cases involving herpesvirus infections. However, the use of antiviral therapy remains a subject of debate, as the benefits are not well established, and treatment decisions often hinge on the clinical presentation and progression of the disease [2,4-6,9].

Once cardiac enzymes demonstrate a decline, arrhythmias are absent, and cardiac systolic function is stabilized, it is recommended that standard HF therapy be continued for a minimum of six months to ensure optimal patient outcomes [7].

## Case presentation

A 34-year-old male with a known history of untreated hypertension presented to the emergency department with a three-day history of lower limb edema, dyspnea on exertion, and pleuritic chest pain. Additionally, the patient reported a cough that had persisted for 10 days.

**Figure 1:**
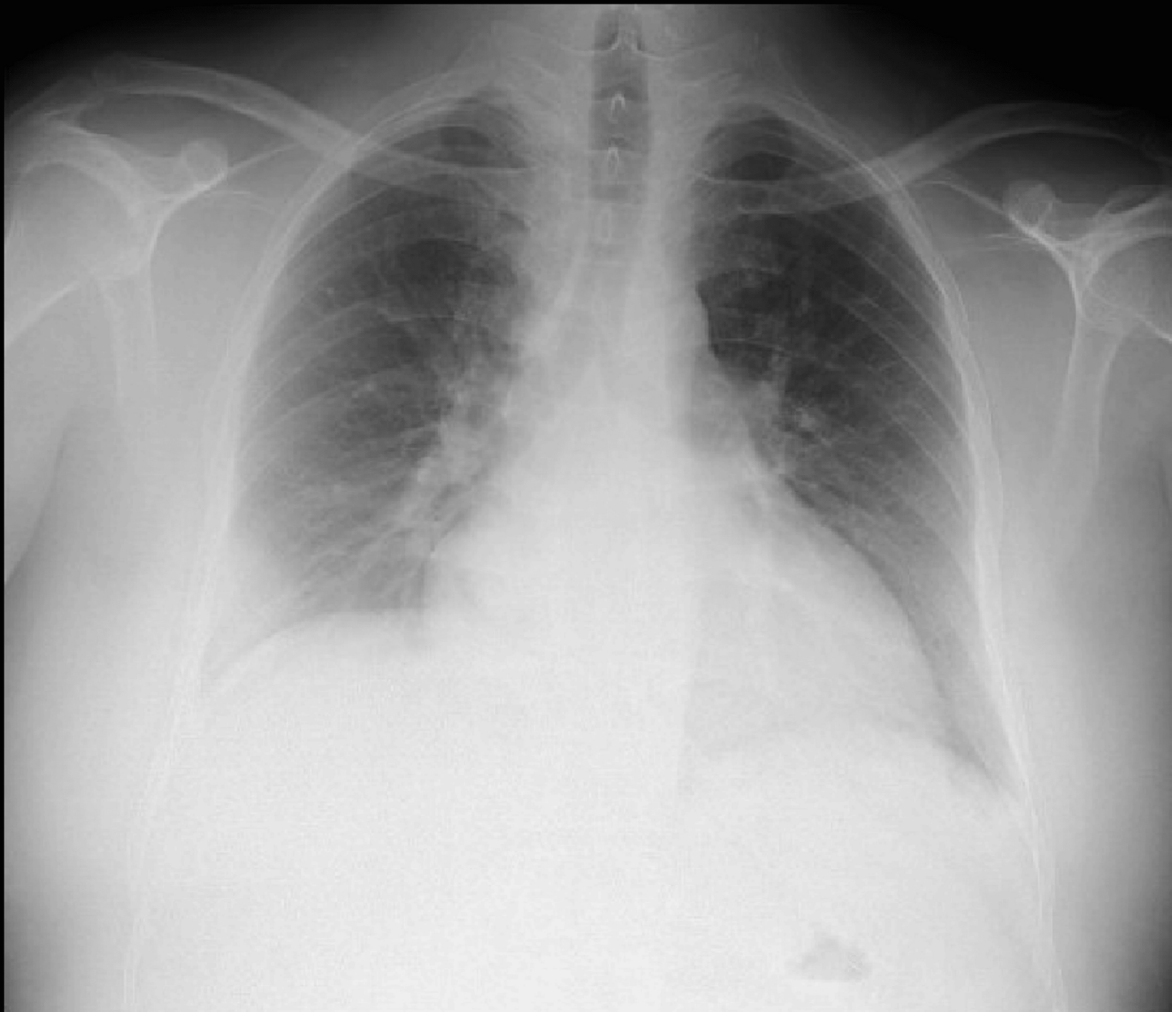
Chest radiography at admission, postero-anterior incidence Demonstrated an increased cardiothoracic index and signs of pulmonary stasis

**Figure 2:**
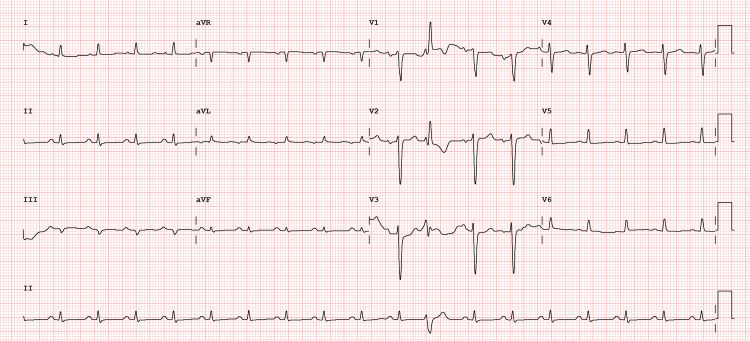
ECG at admission

On physical examination, the patient exhibited signs of rhythmic tachycardia and pulmonary stasis on auscultation, along with peripheral edema extending to the abdominal wall. Laboratory investigations revealed elevated inflammatory markers, significant cytocholestasis, and increased levels of pro-B-type natriuretic peptide (pro-BNP) (Table 1). A chest radiograph demonstrated an increased cardiothoracic index and signs of pulmonary stasis (Figure 1); an electrocardiogram (ECG) showed sinus tachycardia with flattening of T waves (Figure 2). Based on these findings, a diagnosis of de novo HF was made, New York Heart Association (NYHA) classification class III.

**Table 1:** Relevant laboratory investigation results

Laboratory parameter	Result	Reference value	Units
C-reactive protein	8	0.06-1	mg/dL
Alanine transaminase	561	50-136	UI/L
Gamma-glutamyl transferase	301	15-85	UI/L
Total bilirubin	3.92	<1	mg/dL
Pro-B-type natriuretic peptide	6348	<450	pg/mL
Troponin I	1.1	<0.06	ng/mL

**Figure 3:**
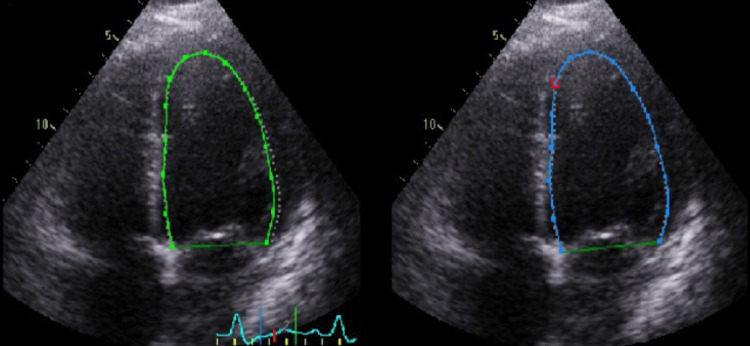
Transthoracic echocardiogram Biventricular dilation. Moderately dilated left ventricle (LVID measured as 63 mm), with globally decreased systolic function (15%) and global hypokinesia. Right ventricle with decreased systolic function (tricuspid annular plane systolic excursion (TAPSE) measured as 14 mm; tricuspid measured as 0.08 m/s). LVID: left ventricular internal dimension

Further inpatient studies revealed elevated troponin I levels (Table 1). Transthoracic echocardiography identified biventricular dilation with a reduced ejection fraction (rEF) of 15% (Figure 3).

**Figure 4:**
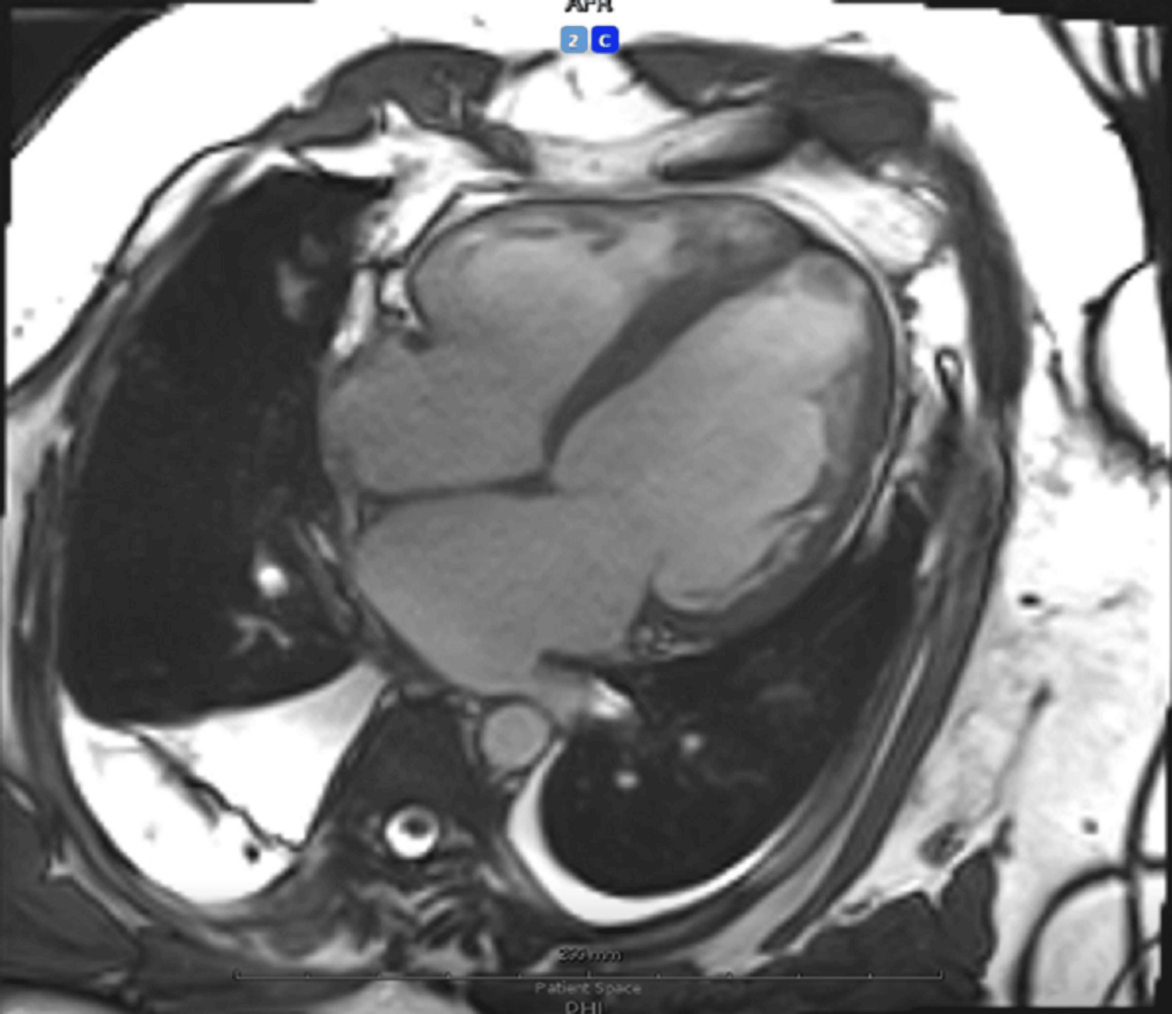
CMRI-4 chamber view showing ventricular and atrial chamber dilation and intracavitary thrombus CMRI: cardiac magnetic resonance imaging

Cardiac magnetic resonance imaging (CMRI), which is the non-invasive reference technique for myocarditis diagnosis [6], showed the left ventricle severely dilated (138 ml/m²) with a significant rEF of 16% due to global hypokinesis. There was the presence of an intracavitary thrombus measuring 12 x 16 mm (Figure 4). Dilation of the left atrium was measured at 55 ml/m². The right ventricle was not dilated (105 ml/m²) and had a compromised ejection fraction (20%). A layer of pericardial effusion was observed. Rest perfusion showed homogeneous myocardial signal enhancement, with transmural delayed enhancement in the apical segment of the septum.

The patient was initiated on standard medical therapy for HF (iACE, beta-blocker, aldosterone antagonist, and diuretics), colchicine for suspected viral myopericarditis, and anticoagulation therapy due to the presence of a left ventricular (LV) thrombus. The initiation of the SGLT2 inhibitor was not implemented, as this case report predates the release of the most recent guidelines that now advocate for its use [7].

This therapeutic approach led to a marked improvement in HF symptoms (NHYA class I), normalization of liver function tests, and a decrease in troponin I levels.

After 14 days, positive serology for herpes simplex virus was identified, with anti-herpes simplex virus type I IgM antibodies detected at twice the upper limit of normal. This finding suggested a viral myocarditis caused by herpes simplex virus infection.

However, given the patient’s clinical and laboratory improvement and after a multidisciplinary meeting involving internal medicine, cardiology, and infectious disease specialists, targeted antiviral therapy was not initiated. The remaining infectious serologies, including cytomegalovirus, Epstein-Barr virus, mononucleosis, Borrelia, hepatitis A, B, and C, adenovirus, and Coxsackie virus, were all negative, as was the autoimmune panel.

At the one-year follow-up, the patient showed significant clinical improvement, classified as NYHA class I. Pro-BNP levels and liver enzymology had normalized. Repeat transthoracic echocardiography demonstrated recovery of systolic function (45%).

## Discussion

This case represents a rare instance of reversible DCM, associated with viral myocarditis, specifically caused by the herpes simplex virus.

The patient initially presented with acute HF and was treated with standard HF management in accordance with established guidelines [2]. Additionally, a short course of colchicine was administered to alleviate symptoms of pericarditis [9].

Despite the presence of positive herpes simplex virus serology, antiviral therapy was withheld following a multidisciplinary decision, given the patient’s clinical and laboratory improvements.

Viral myocarditis is a well-recognized precursor to DCM, but the mechanisms behind its progression from an acute viral infection to chronic cardiomyopathy are complex and multifaceted [4,10]. Understanding the interplay between viral damage and the host’s immune response is crucial for tailoring treatment strategies, as the case demonstrates the nuanced balance between clinical judgment, diagnostic findings, and therapeutic decisions.

Mechanisms of viral myocarditis leading to DCM

The progression from viral myocarditis to DCM involves both direct viral injury to cardiac myocytes and the body’s immune response. In the acute phase of myocarditis, viruses like herpes simplex can directly invade and damage heart cells, causing cell death and inflammation. However, in some cases, the immune system becomes overly active, not only targeting the virus but also attacking the body's own heart tissue in an autoimmune-like reaction [4,10,11]. This sustained immune attack leads to ongoing inflammation and fibrosis, which weakens the heart muscle and contributes to the dilation of the ventricles [10,11].

This patient's case illustrates the typical pathway of myocarditis progressing to DCM, marked by severe systolic dysfunction (with an ejection fraction of 15%) and ventricular dilation observed through echocardiography and CMRI. The identification of an apical thrombus further highlights the severity of the left ventricular impairment, which can occur due to stagnant blood flow in the enlarged, poorly contracting heart chamber [12].

Role of standard HF management

The management of DCM, particularly when secondary to viral myocarditis, largely revolves around established HF treatments [2,5,8,11]. These therapies, including iACE, beta-blockers, aldosterone antagonists, and diuretics, aim to improve cardiac function by reducing the workload on the heart, controlling blood pressure, and mitigating symptoms of fluid overload [2]. The patient’s favorable response to these conventional treatments demonstrates their efficacy in managing the hemodynamic consequences of severe HF, even when viral myocarditis is suspected as the underlying cause.

In addition to standard therapy, this patient was treated with colchicine, an anti-inflammatory agent typically used for acute pericarditis (recommended dosage 0.5 mg twice daily for three months) [13], which was given in this case due to suspected viral myopericarditis. Colchicine has anti-inflammatory properties that may help to dampen the inflammatory response in the myocardium, thereby limiting further myocardial injury and inflammation [13]. However, the precise benefit of colchicine in viral myocarditis remains uncertain, as this is not a widely standardized therapy for the condition, but it is used off-label in certain cases [8,13].

Controversy surrounding antiviral therapy

One of the key points in this case is the decision not to initiate antiviral therapy, despite the detection of herpes simplex virus serology. This decision was made after careful consideration by a multidisciplinary team, which included specialists from internal medicine, cardiology, and infectious disease. The patient had shown significant clinical improvement with HF therapy alone, and both laboratory markers (e.g., normalization of liver enzymes, decrease in troponin I) and imaging findings supported a favorable trajectory.

The role of antiviral therapy in viral myocarditis remains controversial and is not universally recommended [9,11,14]. While antiviral treatment may be beneficial in certain viral infections, there is no consensus on its routine use. The literature shows conflicting results regarding the efficacy of antiviral agents in reversing or preventing the progression of myocarditis to DCM. Schultheiss and colleagues [11] conducted a comprehensive survey evaluating antiviral treatment strategies used for patients with clinically suspected myocarditis and DCM of viral origin. Their findings highlight that, for most antiviral therapies, there is currently no consensus regarding the indications for treatment in these patients. In some cases, antivirals may help reduce viral replication and limit damage to the myocardium, but in other cases, the heart damage is driven more by the host's immune response rather than ongoing viral activity. This makes it challenging to determine whether antiviral therapy will yield clinical benefits in every case [11,15].

Debate over immunosuppressive therapy

Another layer of complexity in managing viral myocarditis lies in the potential use of immunosuppressive therapy.

In cases where the myocarditis is deemed immune-mediated (rather than viral), immunosuppressive treatments like corticosteroids, azathioprine, and intravenous immunoglobulins are often employed to control the inflammatory response.

The systematic review and meta-analysis conducted by Cheng et al. [16] evaluated the efficacy of immunosuppressive therapy in myocarditis over a 30-year period. Most data on lymphocytic myocarditis indicate that treatment typically involves a combined regimen of corticosteroids and a steroid-sparing agent, primarily azathioprine, with mycophenolate mofetil (MMF) being used more recently. In contrast, giant cell myocarditis (GCM) generally requires a more intensive triple therapy regimen that is often cyclosporine-based, combined with corticosteroids and a steroid-sparing agent (azathioprine or MMF). This approach may also include prior induction therapy with agents such as thymoglobulin, muromonab CD3, or rabbit anti-thymocyte globulin. In prospective studies, mortality was slightly lower in the immunosuppressive therapy group compared to the control group (12.5% vs. 18.2%), but the difference wasn't statistically significant (odds ratio: 0.7, 95% CI: 0.3, 1.64). The improvement in left ventricular ejection fraction (LVEF) was also slightly higher in the IS group, though the result showed some variability (95% CI: -2.29, 16.81). In retrospective studies, survival was significantly better in the IS group compared to the control group (hazard ratio: 0.82, 95% CI: 0.69, 0.96). The analysis highlighted that early intervention with immunosuppressive therapy may be beneficial, especially in cases with an autoimmune component or severe inflammatory response.

Overall, the literature supports the use of immunosuppressive therapy as a viable treatment option in managing myocarditis in biopsy-proven virus-negative organ-specific autoimmune lymphocytic myocarditis, though they also call for further research to optimize treatment protocols and identify specific patient populations that would benefit the most [16-19].

In viral myocarditis, the use of immunosuppressive therapy is not recommended. This is due to the risk of suppressing the immune system’s ability to fight the viral infection, which could worsen the disease [17,19]. However, some studies have shown that in certain cases of viral myocarditis, especially when the viral replication is no longer active or when there is evidence of an autoimmune component, immunosuppressive treatments may be beneficial [9].

In this particular case, immunosuppressive therapy was not pursued because there was no strong indication of an autoimmune component, and the patient’s clinical improvement suggested that his immune response had been appropriately modulated by the standard HF treatments and anti-inflammatory measures. The decision-making process here underscores the importance of thorough diagnostic work-up, including viral and autoimmune serologies, which help guide therapy and avoid unnecessary interventions.

Long-term outcomes and follow-up

The prognosis of DCM is influenced by several key factors, including LVEF, the underlying etiology, arrhythmias, and the patient's response to treatment. In general, a reduced LVEF is associated with poorer outcomes, with five-year survival rates ranging from 50% to 70% for patients with moderate to severe DCM. With an annual mortality rate of 5-7% despite optimal medical therapy [20], the primary cause of mortality in DCM patients is pump failure (approximately 70%) due to progressive ventricular dilation, while sudden cardiac death from arrhythmias accounts for the remaining 30% [1].

In a study [21] involving the Trieste Heart Muscle Disease Registry, which analyzed all DCM patients consecutively evaluated from 1988 to 2013, patients were classified based on their LVEF at presentation. Moderately reduced ejection fraction (mrEF) was defined as a baseline LVEF between 40% and 49%, while rEF was defined as an LVEF of <40%. Among the 812 patients enrolled, 22% presented with mrEF at baseline. During a median follow-up of 120 months, the mrEF group showed a significantly lower rate of death or heart transplantation (9% vs. 36%, p < 0.001) and a lower incidence of sudden cardiac death or major ventricular arrhythmias (4.5% vs. 15%, p < 0.001). However, 17% of the mrEF patients progressed to rEF, despite medical therapy, with a restrictive LV filling pattern emerging as the strongest predictor of this progression.

The Intervention in Myocarditis and Acute Cardiomyopathy (IMAC-2) study [22] highlighted that male patients with a LVEF ≤40%, a symptom duration of less than six months, and either myocarditis or acute idiopathic DCM had a worse recovery (measured by the NYHA classification) and a reduced rate of transplant-free survival compared to female patients. This finding mirrors trends seen in the broader HF population [1]. While about 50% of DCM patients experience recovery with symptomatic cardiological therapy in the first 2-4 weeks, approximately 25% will develop persistent cardiac dysfunction [15], reinforcing the importance of early diagnosis and intervention.

In the IMAC-2 study, transplantation-free survival at one, two, and four years was 94%, 92%, and 86%, respectively [22].

Though the recurrence of acute myocarditis is uncommon - only about 1.1% of patients report prior episodes - up to 30% of those with biopsy-confirmed myocarditis will progress to DCM, particularly in patients with severely impaired LVEF [23]. The initial LVEF at presentation is a critical prognostic factor for the risk of transition from acute myocarditis to chronic HF and DCM. In a cohort study [23] of 74 patients with viral myocarditis (mean age: 47 ± 16 years, 66% male, mean LVEF: 51 ± 13%), 27% developed major ventricular arrhythmias, including ventricular tachycardia (VT) and ventricular fibrillation (VF), while 44% experienced polymorphic arrhythmias. The type of arrhythmia is crucial in predicting long-term outcomes. Monomorphic arrhythmias, typically seen in chronic cardiomyopathy, are associated with poor prognosis and an increased risk of major adverse cardiovascular events. In contrast, polymorphic arrhythmias are commonly seen during the acute phase of myocarditis and indicate ongoing systemic infection, which often leads to early adverse outcomes. VT and VF at presentation are independently linked to an elevated risk of sudden cardiac death and other major cardiovascular events [24].

Despite advancements in treatment, DCM often progresses to advanced HF. DCM accounts for over 40% of patients receiving mechanical circulatory support and is the leading cause of heart transplantation, particularly in younger adults (18-39 years) and middle-aged individuals (40-59 years). Notably, DCM is the most common indication for heart transplantation in these age groups, while ischemic heart disease becomes the predominant cause in patients over 60 years. Following transplantation, DCM patients tend to have a favorable long-term prognosis, with a median survival of approximately 12 years due to their younger age and lower burden of comorbidities [25].

In this case, the patient’s significant recovery, with an ejection fraction improving to 45% and symptom relief categorized as NYHA class I, emphasizes the potential for reversibility in cases of viral myocarditis-associated DCM. The favorable outcome also reinforces the critical role of long-term follow-up in managing DCM and viral myocarditis. Serial imaging (such as echocardiography) and biomarker monitoring (like pro-BNP levels) help assess recovery and guide further treatment. In this case, the repeat echocardiogram showing improved systolic function provided objective evidence of the patient’s recovery, corroborating his clinical improvement.

The treatment of chronic HF in DCM should continue indefinitely, even in patients with left ventricular functional recovery, due to a high risk of relapse upon withdrawal of targeted therapies [25].

A pilot, open-label, randomized trial [26] investigated the effects of phased withdrawal of HF medications in patients with DCM who had recovered clinically. The patients included were asymptomatic, had improved LVEF (≥50%), normalized left ventricular end-diastolic volume (LVEDV), and had N-terminal-pro-BNP levels of <250 ng/L. The goal was to assess whether discontinuing HF medications could be safely done in such cases. After six months, those in the continued treatment group underwent treatment withdrawal using the same protocol. A total of 51 patients were enrolled. Over the first six months, 44% of those in the withdrawal group experienced a relapse, compared to none in the continued treatment group (p = 0.0001). After six months, 96% of patients in the continued treatment group attempted treatment withdrawal, and 36% relapsed in the following six months. No deaths occurred in either group, but three serious adverse events were reported in the treatment withdrawal group, including hospital admissions for non-cardiac chest pain, sepsis, and an elective procedure.

The findings indicate that many patients with DCM who have clinically recovered are at risk of relapse following treatment withdrawal. Given that relapse occurred in a significant portion of patients, the study suggests that treatment should be continued indefinitely until more reliable predictors of relapse can be identified [25,26].

Implications for clinical practice

This case underscores the importance of early diagnosis and a tailored, multidisciplinary approach in managing viral myocarditis and DCM. It highlights the evolving understanding of antiviral and immunosuppressive therapies, where more evidence is needed to clarify their roles. While standard HF therapies remain the cornerstone of treatment, each case of viral myocarditis must be evaluated individually, with consideration of the specific viral cause, the patient’s immune response, and their clinical course.

Further research and clinical trials are essential to define when antiviral or immunosuppressive therapies are beneficial. This case contributes to the growing body of evidence suggesting that favorable outcomes can be achieved without antiviral treatment, particularly when there is a robust clinical response to conventional HF management. It also calls for more refined treatment protocols that can better identify which patients will benefit from additional therapies, such as antivirals or immunosuppressants.

## Conclusions

In conclusion, this case highlights the potential for remarkable recovery in patients with DCM secondary to viral myocarditis. It also emphasizes the importance of carefully evaluating the role of antiviral and immunosuppressive therapy in managing DCM related to viral myocarditis. This case underscores the need for further research and clinical trials to clearly define when antiviral treatment is most effective. Ultimately, the successful outcome in this patient underscores the value of a personalized therapeutic approach, supported by multidisciplinary collaboration, in optimizing patient care.
